# Relation Between Brain Morphological Features and Psychiatric Hospitalization Risk in Major Depressive and Bipolar Disorders

**DOI:** 10.1111/acps.13790

**Published:** 2025-02-20

**Authors:** Kamilla W. Miskowiak, Julian Macoveanu, Brice Ozenne, Emily E. Beaman, Vibeke H. Dam, Patrick M. Fisher, Gitte M. Knudsen, Lars V. Kessing, Martin B. Jørgensen, Vibe G. Frokjaer, Anjali Sankar

**Affiliations:** ^1^ Neurocognition and Emotion in Affective Disorders (NEAD) Centre, Mental Health Services, Capital Region of Denmark, and Department of Psychology University of Copenhagen Copenhagen Denmark; ^2^ Psychiatric Centre Copenhagen, Mental Health Services, Capital Region of Denmark Copenhagen Denmark; ^3^ Neurobiology Research Unit Copenhagen University Hospital Rigshospitalet Copenhagen Denmark; ^4^ Department of Public Health, Section of Biostatistics University of Copenhagen Copenhagen Denmark; ^5^ Department of Drug Design and Pharmacology University of Copenhagen Copenhagen Denmark; ^6^ Department of Clinical Medicine University of Copenhagen Copenhagen Denmark

**Keywords:** bipolar disorder, hippocampus, hospitalization, magnetic resonance imaging, magnetic resonance imaging, registry data

## Abstract

**Introduction:**

Patients with mood disorders, especially, major depressive disorder (MDD) and bipolar disorder (BD), are at heightened risk of relapse and psychiatric rehospitalizations. Therefore, there is an urgent need to identify modifiable biomarkers to inform personalized and intensified prevention strategies for those at the greatest risk of relapse and hospital readmissions. Brain structural measures subserving cognitive function hold particular promise among potential predictive biomarkers.

**Methods:**

In the present study, structural magnetic resonance imaging scans were obtained from 319 patients with BD (*n* = 241) or MDD (*n* = 78). [Correction added on 7 March 2025, after first online publication: In the preceding sentence, ‘MDD (n=241) or BD (n=78)’ has been changed to ‘BD (n=241) or MDD (n=78)’.] Longitudinal data on psychiatric hospitalization for up to 10 years were available from the Danish National population‐based registers. Interhemispheric hippocampal asymmetry, a putative marker of cognitive function and brain reserve, was calculated for each patient. The association between hippocampal asymmetry and future psychiatric hospitalization was assessed using a cause‐specific Cox regression model. Exploratory analyses, also using a cause‐specific Cox model, assessed the association of prefrontal and hippocampal gray matter volume and whole‐brain white matter volume with hospitalizations.

**Results:**

The results indicated a negative association between rightward hippocampal asymmetry (i.e., left<right) and risk of future hospitalizations (HR = 0.90, corresponding to a 10‐year risk reduction of 0.018 for a 1% increase in asymmetry, *p* = 0.040). Exploratory analysis indicated that a larger right hippocampus volume was associated with a reduced risk of hospitalization (HR = 0.18, *p* = 0.004) while a larger bilateral dorsolateral prefrontal volume (HR = 1.06, *p* = 0.01) was associated with an increased risk of hospitalization.

**Conclusion:**

The findings suggest a role for hippocampal and, additionally, prefrontal morphological features in the risk of future psychiatric hospitalizations in mood disorders.

**Summary**

- Significant outcomes
  ○ In a sample of 319 individuals with mood disorders, less rightward hippocampal asymmetry and lower right hippocampal volume were linked to an increased risk of future psychiatric hospitalizations, suggesting their role as biomarkers of disease progression.
  ○ Greater DLPFC volume was associated with a higher risk of hospitalization, potentially reflecting compensatory cognitive control mechanisms.
  ○ These findings highlight the importance of neuroimaging in personalized medicine and treatment planning for mood disorders.
- Limitations
  ○ Observational study design, which limits the ability to establish causal relationships.
  ○ Reliance on predefined brain regions of interest, which may exclude other relevant brain‐based biomarkers.

## 1 Introduction

Major depressive disorder (MDD) and bipolar disorder (BD) are significant contributors to the global burden of disease due to their early onset, recurrent nature, and associated functional disability [1]. Despite the availability of treatments, a considerable proportion of patients experience persistent cognitive impairments during otherwise symptom‐free periods [2, 3], with many facing suboptimal levels of functioning, as well as the risk of relapse and psychiatric hospitalization [4, 5]. There is, therefore, a pressing need to identify biomarkers that can facilitate personalized and intensified prevention strategies for those at the greatest risk of relapse and hospitalization. Among the potential predictive biomarkers, cognitive function and neuroimaging measures hold particular promise in guiding such personalized treatment strategies.

Our recent study explored the role of cognitive impairments on the risk for future hospitalization in 518 patients with mood disorders [4]. We found that clinically significant impairments in verbal learning and memory were associated with future psychiatric hospitalizations, even after accounting for mood symptoms and previous hospitalizations [4]. Further, impairment in executive functions was related to poorer educational outcomes. These findings align with previous research, indicating that cognitive impairments are associated with a poorer prognosis [5]. A possible mechanism of this association is that poorer cognition, particularly memory impairment, contributes to difficulties in adhering to treatment, such as managing medication schedules and recalling therapy information and appointments. Such noncompliance exacerbates mood symptoms and may elevate suicide risk, a leading cause of psychiatric hospital admissions in patients with mood disorders. However, in contrast with the growing evidence for the importance of cognition, there remains a scarcity of evidence regarding brain‐based biomarkers predictive of prognosis.

The hippocampus emerges as a promising prognostic neurocircuitry biomarker due to its crucial role in learning and memory and its commonly observed volume reduction across neuropsychiatric disorders [6]. Other candidate neurocircuitry markers, such as dorsolateral prefrontal cortex (DLPFC) volume, anterior cingulate cortex (ACC) volume, and white matter integrity and volume, have been associated with levels of cognitive function [7, 8] and treatment success in mood disorders [9, 10], albeit with some variability. Specifically, hippocampal pathology and volume reduction are believed to underlie patients' verbal learning and memory deficits [6], which have direct adverse effects on their occupational functioning [11] and prognosis of illness [4]. The prevailing hypothesis posits that a decrease in hippocampal volume, indicative of reduced neuroplasticity and neurogenesis, plays a central role in verbal memory impairments observed not only in mood disorders but also in conditions like mild cognitive impairment (MCI) and Alzheimer's disease [6]. Conversely, augmentation of hippocampal volume may be a significant indicator of treatment response to various treatments targeting cognition, including EPO [12, 13] and lithium [14] in mood disorders.

An interesting but less known putative biomarker with prognostic value is interhemispheric hippocampal asymmetry. The healthy brain typically exhibits rightward (left<right) hippocampal asymmetry, which is considered a marker of brain health or reserve [15]. In contrast, patients with MCI show little hemispheric hippocampal asymmetry, while patients with type 2 diabetes or Alzheimer's disease exhibit the opposite pattern of asymmetry, particularly those with two (relative to one or zero) copies of the apolipoprotein E (ApoE) ɛ4 allele, a well‐established Alzheimer's risk genotype [16]. Consequently, the loss of interhemispheric hippocampal asymmetry has emerged as a key contributor to cognitive decline and putative biomarker across various neurologic and neuropsychiatric conditions, including type 2 diabetes [17] carriage of the ApoE ɛ4 allele [18], as well as in BD [19] and MDD [20].

Limitations of previous studies include their cross‐sectional design and generally modest sample sizes (*n* < 80). The present study addresses these limitations by being the largest neuroimaging investigation to date with longitudinal data on psychiatric hospitalizations, involving > 300 participants with mood disorders, followed up through the Danish Registers for up to 10 years. The primary aim of this study is to investigate whether less rightward (or larger left vs. right hippocampal volume ratio) hippocampal asymmetry (as an indicator of lower brain reserve) is associated with future psychiatric hospitalization. Secondary exploratory analyses aim to examine the associations of hippocampal volume, DLPFC volume, ACC volume, and white matter volume, with future psychiatric hospitalization.

## 2 Materials and Methods

### 2.1 Participants

**FIGURE 1:**
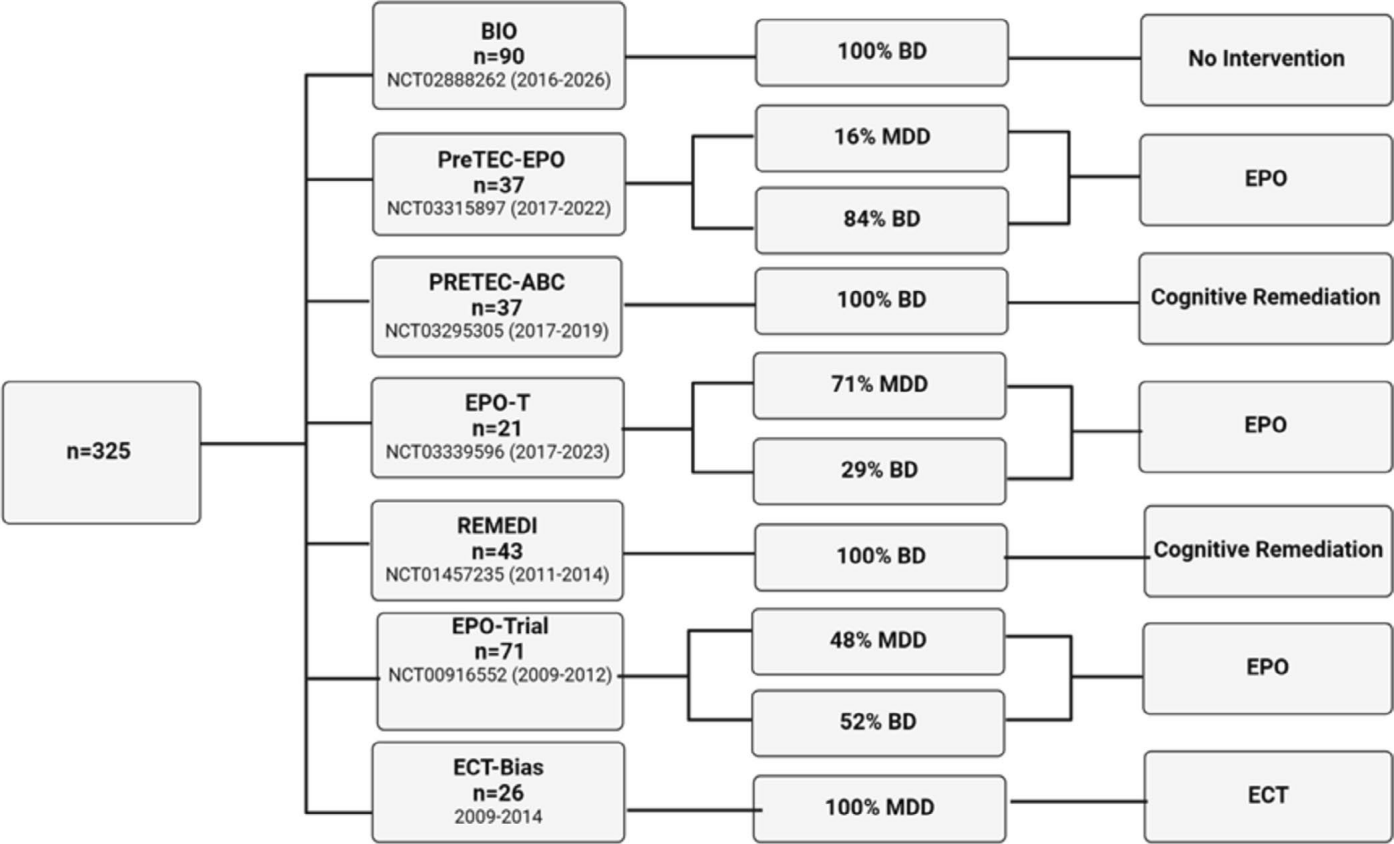
Sample selection flowchart. Figure 1 provides details of the seven clinical studies from which demographic, clinical, and MRI data from patients with major depressive disorder and bipolar disorder were used for this study. All studies except one (BIO) were intervention trials; however, only pretreatment demographic, clinical, and MRI data were used in the estimation of risk of future psychiatric hospitalizations.

The present study included 325 patients who met the criteria for either BD (*n* = 244) or MDD (*n* = 81), according to the International Classification of Disorders (ICD‐10), assessed using the Schedule for Clinical Assessment in neuropsychiatry (SCAN) structured clinical interview [21]. The patients with affective disorders (i.e., BD or MDD) were recruited between 2009 and 2019 from the Copenhagen Affective Disorder Clinic, community psychiatric centers, private clinics, and general practices. The patients recruited between 2009 and 2019 participated in one of seven trials, of which six were intervention studies (Figure 1). In the present study, only baseline (or pre‐treatment) neuroimaging data from the intervention studies were used. Of the 81 patients with MDD, 42% additionally met the criteria for treatment resistance depression (TRD) based on the assessment of their medical treatment history with the Treatment Response to Antidepressants Questionnaire. Exclusion criteria for all patients were a diagnosis of schizophrenia or schizoaffective disorder, current (i.e., within the past three months of inclusion) alcohol or substance use disorder, and acute suicidal risk (score > 2 on the suicide item of the 17‐item Hamilton Depression Rating Scale). Additional exclusion criteria applied to a subpopulation of patients (*n* = 129) who were recruited to participate in the erythropoietin trials included significant medical conditions (diabetes, renal failure, epilepsy, hypertension, present or past malignancies, and thromboses), pregnancy or breastfeeding, contraceptive medication, smoking, BMI greater than 30 kg/m^2^, and body weight less than 45 kgs or more than 95 kgs.

Written informed consent was obtained from all participants before their inclusion in the respective clinical trials, and letters were sent to their general practitioners to rule out a history of significant medical conditions. All procedures were performed in accordance with the Capital Region of Denmark Ethical Committee standards (more information in the section S1).

### 2.2 Procedure

#### 2.2.1 Structural MRI

Structural MRI data were acquired at two sites. Between 2009 and 2014, scans were performed at the Danish Research Centre for Magnetic Resonance (DRCMR; *n* = 140) on a 3 Tesla Siemens Trio MR scanner with an 8‐channel head array coil. T1‐weighted structural images were acquired using a 3D MPRAGE sequence with the following parameters: inversion time: 800 ms, echo time (TE): 3.93 ms, repetition time (TR): 1540 ms, flip angle = 9°, field of view (FOV): 256 × 256, 192 slices. Between 2017 and 2019, scans (*n* = 185) were performed at the Copenhagen University Hospital (Rigshospitalet) using a 3 Tesla Siemens Prisma scanner (Siemens, Erlangen, Germany) with a 64‐channel head–neck coil. T1‐weighted structural images were acquired using a 3D MPRAGE sequence with the following parameters: TE = 2.58 ms, TR = 1900 ms, flip angle = 9°, FOV = 230 × 230 mm, slice thickness = 0.9 mm.

All T1‐weighted images were processed using FreeSurfer analysis suite v7.3.2 (http://surfer.nmr.mgh.harvard.edu/) using the default workflow. In short, the images were skull‐stripped, intensity‐normalized, transformed to Talairach space, and automatically segmented to extract gray and white matter components and subcortical structures like the hippocampi [22]. The total intracranial volume (TIV) was also computed and used to control the effect of individual's brain size during the statistical analysis. The accuracy of the computed cortical surfaces was assessed using the ENIGMA Cortical Quality Control Protocol 2.0 [23]. Detected errors in the cortical reconstructions were subsequently corrected manually.

#### 2.2.2 Regions of Interest

Based on our a priori hypotheses, the Hippocampus Asymmetry Index (HAI) was the primary neuroimaging measure of interest. HAI was calculated for each person as done previously by us and others using the formula below:

$$\mathrm{HAI}=\left(\frac{\text{Right hippocampus volume}-L\text{eft hippocampus volume}}{\text{Total hippocampus volume}}\right)*100$$

**FIGURE 2:**
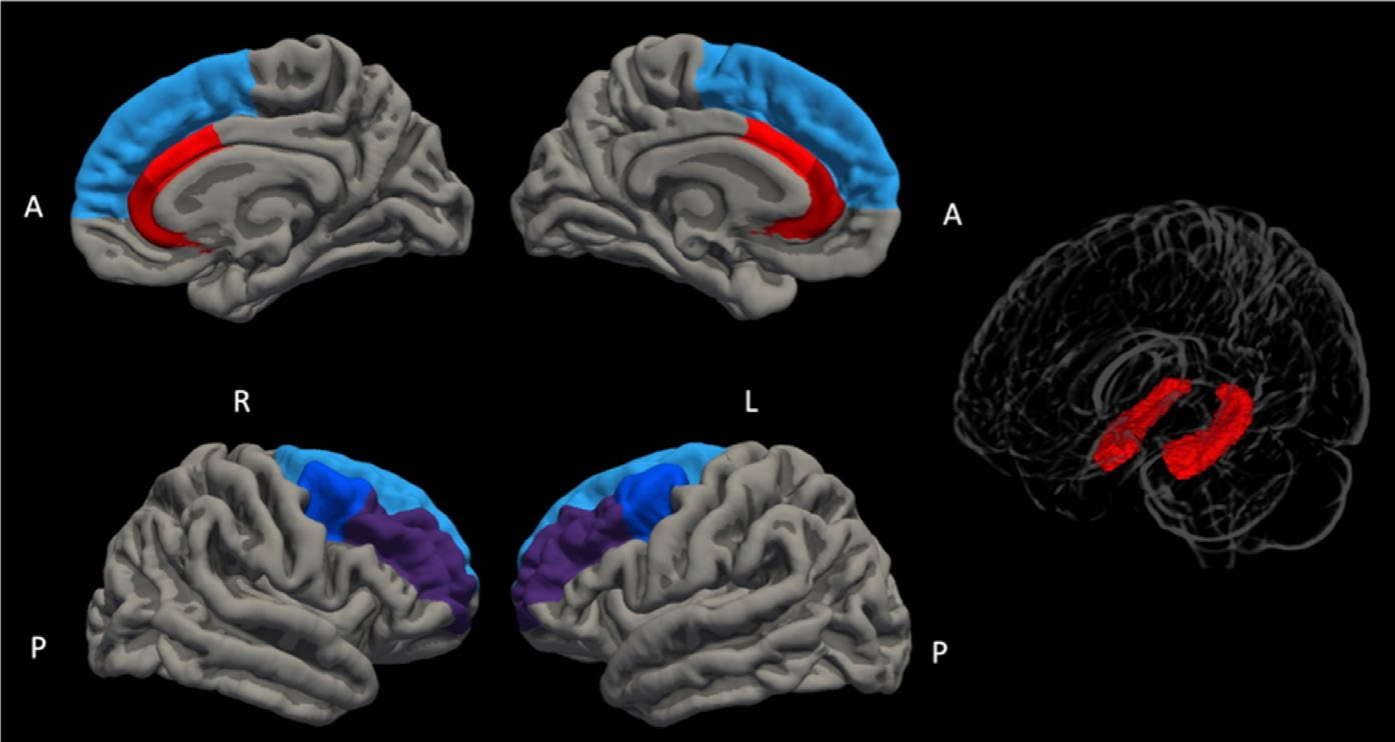
Bilateral hippocampus, dorsolateral prefrontal cortex, and anterior cingulate cortex regions of interest. Cortical regions are based on the Desikan–Killiany Atlas and the hippocampus region on the Harvard–Oxford Subcortical Structural Atlas.

The left and right hippocampi were delineated using the FreeSurfer automated volumetric segmentation pipeline. For the exploratory analyses, the following regions of interest were included: left and right hippocampus, bilateral DLPFC, bilateral ACC, and total white matter volume. The bilateral DLPFC region of interest was constructed by adding the left and right caudal and rostral middle frontal and superior frontal regions from the automated cortical parcellation of the Desikan–Killiany atlas. The bilateral ACC region of interest was constructed by adding the left and right caudal and rostral ACC regions from the automated cortical parcellation of the Desikan–Killiany atlas (Figure 2).

#### 2.2.3 Mood Ratings

Mood symptom severity was assessed on the day of inclusion using the Hamilton Depression Rating Scale 17 items (HDRS‐17) [24] and, additionally, using the Young Mania Rating Scale (YMRS) [25] for patients with BD.

#### 2.2.4 The Danish National Registers

The demographic, clinical, and structural MRI data from the participants were linked to Danish population‐based registry data using the unique personal identification number assigned to all persons living in Denmark since 1968. More information on the Danish National Registers is detailed in section S2. The primary outcome was psychiatric hospitalization in the follow‐up period since inclusion in the study (i.e., acquisition of structural MRI). Information on psychiatric hospitalization was available from the Danish National Patient Register and the Danish Psychiatric Central Research Register of the National Registers. Psychiatric hospitalization was defined as admission to a psychiatric hospital as an inpatient (patients admitted during daytime or overnight to a psychiatric hospital) with at least one main diagnosis of MDD, mania, or BD (ICD‐10 codes F30, F31, F32, F33, F38, and F39). Hospitalization records were available until 2019, which marked the end of the follow‐up period.

#### 2.2.5 Statistical Analyses

**FIGURE 3:**
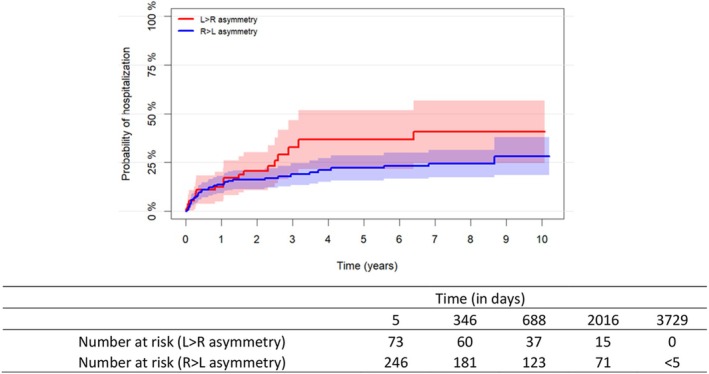
Aalen–Johansen survival estimates of the probability of being hospitalized in individuals with rightward (R>L) and leftward (L>R) hippocampal asymmetry. The plot illustrates the probability of getting hospitalized in patients with bipolar disorder or major depressive disorder in individuals with L>R hippocampal asymmetry and in those with R>L hippocampal asymmetry. Asymmetry was categorized as L>R and R>L only for visualization purposes, while in the Cox model, hippocampal asymmetry was included as a continuous variable. The risk was estimated using the Aalen–Johansen estimator. The y‐axis represents the cumulative risk of hospitalization, and the x‐axis represents time in years.

The risk of hospitalization was estimated for right>left HAI and left>right HAI using the Aalen–Johansen estimator (Figure 3) [26]. Hippocampal asymmetry was categorized as a binary variable (i.e., right>left and left>right) for visualization purposes only. In all the analyses performed herein, hippocampal asymmetry was included as a continuous variable based on the HAI formula noted above.

The primary analysis of the relationship between rightward hippocampal asymmetry and psychiatric hospitalization was modeled using cause‐specific Cox regression, with the event being psychiatric hospitalization or death, and censoring on emigration (*n* = 8), or end of the follow‐up period, whichever came first. The cause‐specific Cox regression model relative to psychiatric hospitalization was adjusted for age, sex, hospitalization in the year preceding inclusion, HDRS‐17 scores, diagnosis (i.e., BD vs. MDD), year of inclusion, intracranial volume, and the clinical trial from which the participant was recruited. MR scanner was not included as a covariate as it is not predictive of psychiatric hospitalization and, furthermore, as it is related to the year of inclusion (2009–2014 performed in DRCMR scanner, while 2017–2019 performed in Rigshospitalet scanner). Splines were included to account for a possible non‐linear relationship between age and the log hazard of hospitalization. Because death was very rare in this sample (*n* < 5), the cause‐specific Cox model relative to death did not include any covariates. Therefore, the cause‐specific hazard ratio (HR) will only always refer to the Cox model with respect to psychiatric hospitalization. The 10 ‐year‐risk presented in Table 2 was evaluated based on the average predicted risks from the cause‐specific Cox models. Patients with BD were either euthymic or in a depressive episode and had low scores on the YMRS (see Table 1), and therefore, YMRS scores were not included in the model. As HDRS‐17 scores were missing for 6 patients, the final sample used for analysis included 319 patients with BD or MDD. The results will be reported for the final sample of 319 patients. In addition to the cause‐specific Cox model, a sensitivity analysis was performed to test the robustness of the results to the proportional hazard assumption using the inverse probability of censoring weighting (IPCW) function implemented in the risk Regression package in R.

**TABLE 1:** Baseline demographic and clinical information.

	Patients (*n* = 319)
Baseline demographic information	
Age (mean ± SD)	36.1 ± 11.0
Gender (% female)	65.5%
Clinical information	
BD/MDD (*N*)	241/78
HDRS‐17 (mean ± SD)	10.3 ± 8.9
YMRS (mean ± SD)^a^	2.6 ± 3.1
Future hospitalizations (*N*)	66
Hospitalizations in the year preceding inclusion (*N*)	80

Abbreviations: %: percentage; BD: Patients with bipolar disorder; HDRS‐17: Hamilton Depression Rating Scale 17 items; MDD: Patients with major depressive disorder; *N*: total number; SD: standard deviation; YMRS: Young Mania Rating Scale.

^a^ YMRS was administered only to patients with BD.

Exploratory analysis examined whether left and right hippocampus volume, bilateral DLPFC volume, bilateral ACC volume, and total white matter volume were associated with the risk of hospitalization. As done for primary analysis, the Cox regression model relative to psychiatric hospitalization was adjusted for age (using p‐spines), sex, hospitalization in the year preceding inclusion, HDRS‐17 scores, diagnosis (i.e., BD vs. MDD), year of inclusion, intracranial volume, and the clinical trial from which the participant was recruited. Smoothed splines were included in all the models to account for any nonlinear relationship between age and log hazard of hospitalization.

For 281 patients, information on the number of previous depressive episodes, an index of illness chronicity, and verbal learning and memory scores was available. We included verbal learning and memory as an additional covariate as it is associated with hippocampal volume [6] and, additionally, as we have previously shown that low verbal learning and memory is associated with an increased risk of psychiatric hospitalization in an overlapping sample [4]. Thus, post hoc analyses were performed in this subgroup of patients covarying for both the number of depressive episodes and verbal learning and memory scores. The number of depressive episodes was dichotomized as patients with ≤ 5 previous episodes or > 5 previous episodes. Further information on the neuropsychological tests and thresholds used in the calculation of verbal learning memory scores is detailed in section S3.

## 3 Results

The sample consisted of 319 patients with affective disorders [ages 18–65 years, mean age ± standard deviation (SD) = 36.1 ± 11.0 years; 65.5% female]. Patients with BD (*n* = 241) were either euthymic or in a depressive episode at inclusion (HDRS‐17 score, mean ± SD: 6.7 ± 5.8) and thus had low scores on the YMRS (mean ± SD: 2.6 ± 3.1). Patients with MDD (*n* = 78) were in a depressive episode at inclusion (HDRS‐17 mean ± SD: 21.3 ± 7.8). Data on psychiatric hospitalization until 2019 were available for all patients. Sixty‐six patients had at least one record of postassessment psychiatric hospitalization, while 80 patients had a record of psychiatric hospitalization in the year preceding psychiatric hospitalization. The baseline demographic and clinical information of the sample are tabulated in Table 1.

The cause‐specific Cox models estimated a negative association between rightward hippocampal asymmetry and the hazard of future hospitalizations (HR = 0.90, 95% CI:0.82–1.00, *p* = 0.040; Table 2). In terms of risk, the cause‐specific Cox model estimated an average 10‐year risk of hospitalization of 27.61% in the population. If every patient had a reduction in rightward hippocampal asymmetry by 0.01, this average risk would have been 25.73%, that is, 1.88% lower risk of psychiatric hospitalization for a 1% increase in asymmetry. A list of covariates significantly associated with the risk of hospitalizations and corresponding HRs is presented in text S4.

**TABLE 2:** Risk quantification for the structural MR biomarkers.

Biomarker	Quantile (q25, q50, q75)	Hazard ratio [95% CI], *p*	10‐year risk comparison
Hippocampal asymmetry (%)	0.067, 1.84, 3.60	HR = 0.90 [0.82–1.00], *p* = 0.040	27.61–25.73 = 1.88% decrease
			For a 1% asymmetry increase
Right hippocampal volume (cubic cm)	4.13, 4.41, 4.73	HR = 0.18 [0.06–0.57], *p* = 0.004	28.02–25.02 = 3.0% decrease
			For a 0.1 cubic cm increase
Left hippocampal volume (cubic cm)	4.95, 5.36, 5.56	HR = 3.06 [0.90–10.43], *p* = 0.08	28.02–30.06 = 2.04% increase
			For a 0.1 cubic cm increase
Bilateral DLPFC volume (cubic cm)	86.19, 93.40, 103.02	HR = 1.06 [1.01–1.11], *p* = 0.01	28.02–29.08 = 1.06% increase
			For a 1 cubic cm increase
Bilateral ACC volume (cubic cm)	8.05, 9.07, 9.98	HR = 0.92 [0.72–1.17], *p* = 0.49	28.02–27.86 = 0.16% decrease
			For a 0.1 cubic cm increase
WM volume (cubic cm)	442.45, 476.26, 514.80	HR = 1.00 [0.99–1.00], *p* = 0.79	28.02–28.23 = 0.21% increase
			For a 10 cubic cm increase

*Note*: The first column represents the list of structural MR biomarkers tested in this study. The 25% (q25), median (q50), and 75% (q75) values of the biomarker in the sample are presented in the second column. The hazard ratio (HR) with respect to psychiatric hospitalization for each biomarker is presented in the third column. The fourth column represents the average risk observed in the sample vs. the average risk that would have been observed had every individual had an increase (unit increase reported in the column) in biomarker value. Please note that the risk change cannot be compared between biomarkers since they do not correspond to comparable biomarker increases.

Post hoc analysis (*n* = 281; 58 instances of future psychiatric hospitalizations) also indicated that the inclusion of the number of depressive episodes and verbal learning and memory as covariates to the model did not alter the hazard ratio related to the association between rightward hippocampal asymmetry and future hospitalizations (HR: 0.90, 95% CI:0.81–1.01). Furthermore, the results of the sensitivity analyses, relaxing the proportional hazard assumption, also showed that rightward hippocampal asymmetry was negatively associated with 3‐ to 8‐year risk of psychiatric hospitalization (odds ratio ranging between 0.86 and 0.81 and p‐value between 0.02 and 0.13, see Table S1).

Exploratory analyses revealed that a larger bilateral DLPFC volume (HR = 1.06, 95% CI:1.01–1.11, *p* = 0.01; Table 2) was associated with an increased risk for hospitalizations, while a larger right hippocampus volume was associated with a reduced risk of hospitalization (HR = 0.18, 95% CI:0.06–0.57, *p* = 0.004). Left hippocampal volume (HR = 3.06, 95% CI: 0.90–10.43, *p* = 0.08), bilateral ACC volume (HR:0.92 95% CI: 0.72–1.17, *p* = 0.49), and white matter volume (HR:1.00 95% CI: 0.99–1.00, *p* = 0.79) were not significantly associated with the risk of future hospitalizations. A list of covariates significantly associated with the risk of hospitalizations and corresponding HRs is presented in text S4.

Post hoc analysis (*n* = 281; 58 instances of future hospitalizations) indicated that the inclusion of the two additional covariates (i.e., number of depressive episodes and verbal learning and memory) to the model did not alter much the hazard ratio related to the association among the bilateral DLPFC volume (HR = 1.06, 95% CI:1.01–1.11), right hippocampal volume (HR = 0.20, 95% CI:0.06–0.71), and future hospitalizations.

## 4 Discussion

This neuroimaging study investigates hippocampal asymmetry as a prognostic marker for clinical outcomes in mood disorders. Consistent with the primary hypothesis, lower rightward hippocampal asymmetry was associated with future psychiatric hospitalizations in our cohort of 319 patients with BD or MDD, who were followed for up to 10 years. Further, a smaller right hippocampal volume and larger DLPFC volume, but not white matter volume, were significantly associated with future psychiatric hospitalizations.

The observation that patients with less rightward hippocampal asymmetry exhibited a greater risk of future psychiatric hospitalizations is consistent with evidence for hippocampal asymmetry being a neurocircuitry biomarker of ‘brain reserve’ that may be affected across several neuropsychiatric disorders, including BD [19], MDD [20], MCI, Alzheimer's disease [16, 18], and type 2 diabetes [17]. Brain reserve refers to a person's total neurobiological resources at a given point in time [27]. Notably, the observed association in our study was significant even after controlling for differences in illness chronicity, prior hospitalizations, mood symptom severity, demographic variables, as well as verbal learning and memory scores, which we have previously found to be associated with increased risk of psychiatric hospitalizations in an overlapping sample [4]. Our finding, therefore, provides crucial evidence, supporting the idea that changes in a hippocampal structure may influence the course of mood disorders, affecting susceptibility to relapse and hospitalization.

The other identified neurocircuitry risk markers were a smaller right hippocampal volume and a larger bilateral DLPFC volume. The association between lower right hippocampal volume and subsequent hospitalizations aligns with previous findings, indicating hippocampal volume reductions across a spectrum of neuropsychiatric conditions, including mood disorders [28], schizophrenia [29], and Alzheimer's [6]. Notably, interventions targeting cognition and mood symptoms, such as EPO [12, 13] and lithium [14], have been linked to hippocampal volume augmentation. This dual relationship suggests that hippocampal volume alterations may serve as biomarkers for disease progression and cognitive enhancement, respectively. Consequently, they offer a crucial and much‐needed tool for prognostication and treatment selection aimed at cognitive improvement [6].

The association between *greater* DLPFC volume and the risk of hospitalizations may at first seem counterintuitive. We speculate that this association could suggest the involvement of compensatory cognitive control mechanisms in disease progression. While many studies on mood disorders have reported cortical thinning or reduced volume of the DLPFC [30, 31], our prior research identified increased cortical thickness in remitted patients experiencing cognitive impairments compared to those without cognitive deficits [32]. This augmented thickness of the DLPFC in cognitively impaired patients was interpreted as ongoing compensatory neurocognitive mechanisms [32]. Within this framework, a larger DLPFC volume might signal sustained long‐term efforts to maintain symptom stability, which could potentially falter during periods of significant adversity, thereby serving as an indicator of susceptibility to future hospitalizations. Alternatively, greater DLPFC volume could reflect abnormalities in synaptic pruning during neurodevelopment, leading to less refinement of neural circuits. For instance, studies indicate dysfunctional synaptic pruning mechanisms in psychotic disorders like schizophrenia [33], which share certain neurodevelopmental underpinnings with mood disorders, including both excessive and insufficient PFC plasticity [34, 35]. However, limited evidence exists regarding synaptic pruning abnormalities specifically in mood disorders.

The hippocampal asymmetry finding provides a key structural correlate for the previously observed association between verbal memory impairment and future hospitalization risk in an overlapping larger cohort [4]. The underlying pathophysiological processes for the association of lower rightward hippocampal asymmetry and volume in clinical outcomes likely involve neuroplasticity deficits and disrupted stress response systems. Indeed, converging evidence from postmortem studies in mood disorders indicates that memory impairment stems from disrupted neuroplasticity and structural changes in key neurocircuitries, including the hippocampus [36]. Chronic stress, a common feature in mood disorders, has been implicated in hippocampal volume reductions and alterations in hippocampal asymmetry [37]. Preclinical research implicated chronic stress‐related glucocorticoid overexposure in dendritic retraction, hippocampal pyramidal cell death, and suppressed dentate gyrus neurogenesis [38, 39, 40]. Postmortem examination of hippocampal tissue from patients with MDD corroborates these findings, showing loss of dendritic branching and spine complexity, but no suppressed neurogenesis [38, 40]. Together, this evidence supports the potential efficacy of interventions targeting neuroplasticity to alleviate memory impairment and improve long‐term prognosis in mood disorders.

While the observational study design limits the ability to establish causal relationships, the longitudinal design and control for various illness variables provided significant insights, supporting the predictive value of the identified brain‐based biomarkers. However, a limitation of this study is the reliance on predefined brain regions of interest, which may exclude other relevant brain‐based biomarkers.

Our sample comprised patients with both BD and MDD, who were overall—at a group level—in full or partial remission, but with MDD participants exhibiting moderate depression severity at baseline. Despite this heterogeneity, baseline diagnosis and depressive symptoms did not significantly influence the observed risk of future hospitalizations. There was variability in illness chronicity among our sample. However, post hoc covariation for the number of prior depressive episodes did not substantially alter the associations between lower rightward hippocampal asymmetry, lower right hippocampal volume, larger DLPFC volume, and future psychiatric hospitalizations. Approximately 25% of patients had been hospitalized before their baseline assessment, a known predictor of future psychiatric hospitalization. Nonetheless, analyses were adjusted for prior hospitalizations in the year preceding the baseline assessment, indicating that the association between less rightward hippocampal asymmetry and future hospitalizations is significant even after controlling for prior hospitalizations.

## 5 Conclusion

In conclusion, our findings contribute to the growing body of evidence implicating neurocircuitry biomarkers in the prediction of clinical outcomes in mood disorders. The identification of hippocampal asymmetry, along with hippocampal and DLPFC volume, as potential prognostic markers underscores the importance of neuroimaging‐based approaches in personalized medicine and treatment planning for patients with mood disorders. Notably, we and others have previously shown that these regions show structural changes following procognitive behavioral and pharmacological interventions [6, 12, 41]. Future research endeavors should aim to elucidate the underlying mechanisms linking neurocircuitry alterations to disease progression and develop targeted interventions that enhance neuroplasticity to mitigate adverse clinical outcomes in this vulnerable population.

## Author Contributions

Professor Miskowiak made substantial contributions to the conception and design of the study, interpretation of findings, drafting of the manuscript, and critical revisions for intellectual content. Dr. Macoveanu substantially contributed to data curation and analysis, interpretation of findings, and critical revisions of the manuscript. Dr. Ozenne provided statistical expertise for all analyses performed herein and substantially contributed to the interpretation of findings and critical revisions of the manuscript. Ms. Beaman and Dr. Dam substantially contributed to data curation and critical revisions of the manuscript. Dr. Fisher, Professor Knudsen, Professor Kessing, Professor Jørgensen, and Professor Frokjaer substantially contributed to the interpretation of findings and critical revisions of the manuscript. Dr. Sankar made substantial contributions to the design of the study, analysis of data, interpretation of findings, and drafting and critical revisions of the manuscript.

## Conflicts of Interest

Dr. Miskowiak has received consultancy fees from Lundbeck, Janssen, and Angelini Pharma in the past three years. Dr. Knudsen has within the last three years served as a consultant for Onsero, Gilgamesh, Pure, Pangea, and Sanos and as a speaker for Abbvie, Angelini, and H. Lundbeck. Dr. Frokjaer has served as a consultant for SAGE therapeutics, and lecturer for H. Lundbeck, Janssen‐Cilag, and Gedeon‐Richther. Dr. Dam has served as a lecturer for H. Lundbeck. Dr. Madsen has served as a lecturer for the Lundbeck Foundation and H. Lundbeck. All other authors declare no conflicts of interest.

### Peer Review

The peer review history for this article is available at https://www.webofscience.com/api/gateway/wos/peer‐review/10.1111/acps.13790.

## Supporting information


Data S1.

## Data Availability

Deidentified data are available from the Cimbi database to researchers via email to cimbi@cimbi.dk. As per National Health Registry regulations, access to register data needs prior approval, and this can be obtained from Statistics Denmark's research services (https://www.dst.dk/en/TilSalg/Forskningsservice/Dataadgang).

## References

[1] H. A. Whiteford , L. Degenhardt , J. Rehm , et al., “Global Burden of Disease Attributable to Mental and Substance Use Disorders: Findings From the Global Burden of Disease Study 2010,” Lancet 382 (2013): 1575–1586. 23993280 10.1016/S0140-6736(13)61611-6

[2] K. W. Miskowiak , H. L. Kjaerstad , C. K. Lemvigh , et al., “Neurocognitive Subgroups Among Newly Diagnosed Patients With Schizophrenia Spectrum or Bipolar Disorders: A Hierarchical Cluster Analysis,” Journal of Psychiatric Research 163 (2023): 278–287. 37244066 10.1016/j.jpsychires.2023.05.025

[3] J. H. Jensen , U. Knorr , M. Vinberg , L. V. Kessing , and K. W. Miskowiak , “Discrete Neurocognitive Subgroups in Fully or Partially Remitted Bipolar Disorder: Associations With Functional Abilities,” Journal of Affective Disorders 205 (2016): 378–386. 27573491 10.1016/j.jad.2016.08.018

[4] A. Sankar , S. C. Ziersen , B. Ozenne , et al., “Association of Neurocognitive Function With Psychiatric Hospitalization and Socio‐Demographic Conditions in Individuals With Bipolar and Major Depressive Disorders,” EClinicalMedicine 58, no. 58 (2023): 101927, 10.1016/j.eclinm.2023.101927. 37007740 PMC10050788

[5] H. L. Kjaerstad , T. Haldorsen , M. Vinberg , L. V. Kessing , and K. W. Miskowiak , “Associations Between Emotional and Non‐emotional Cognition and Subsequent Mood Episodes in Recently Diagnosed Patients With Bipolar Disorder: A 16‐Month Follow‐Up Study,” Journal of Affective Disorders 324 (2023): 16–23. 36565963 10.1016/j.jad.2022.12.061

[6] C. V. Ott , C. B. Johnson , J. Macoveanu , and K. Miskowiak , “Structural Changes in the Hippocampus as a Biomarker for Cognitive Improvements in Neuropsychiatric Disorders: A Systematic Review,” European Neuropsychopharmacology 29 (2019): 319–329. 30654916 10.1016/j.euroneuro.2019.01.105

[7] J. L. Jorgensen , J. Macoveanu , J. Z. Petersen , et al., “Association of Childhood Trauma With Cognitive Impairment and Structural Brain Alterations in Remitted Patients With Bipolar Disorder,” Journal of Affective Disorders 337 (2023): 75–85. 37236273 10.1016/j.jad.2023.05.078

[8] J. Zarp petersen , C. Varo , C. F. Skovsen , et al., “Neuronal Underpinnings of Cognitive Impairment in Bipolar Disorder: A Large Data‐Driven Functional Magnetic Resonance Imaging Study,” Bipolar Disorders 24 (2022): 69–81. 33955648 10.1111/bdi.13100

[9] M. B. Mogensen , J. Macoveanu , G. M. Knudsen , C. V. Ott , and K. W. Miskowiak , “Influence of Pre‐Treatment Structural Brain Measures on Effects of Action‐Based Cognitive Remediation on Executive Function in Partially or Fully Remitted Patients With Bipolar Disorder,” European Neuropsychopharmacology 56 (2022): 50–59. 34933219 10.1016/j.euroneuro.2021.11.010

[10] I. Seeberg , H. L. Kjaerstad , and K. W. Miskowiak , “Neural and Behavioral Predictors of Treatment Efficacy on Mood Symptoms and Cognition in Mood Disorders: A Systematic Review,” Frontiers in Psychiatry 9 (2018): 337. 30093870 10.3389/fpsyt.2018.00337 PMC6071514

[11] S. Tse , S. Chan , K. L. NG , and L. N. Yatham , “Meta‐Analysis of Predictors of Favorable Employment Outcomes Among Individuals With Bipolar Disorder,” Bipolar Disorders 16 (2014): 217–229. 24219657 10.1111/bdi.12148

[12] K. W. Miskowiak , M. Vinberg , J. Macoveanu , et al., “Effects of Erythropoietin on Hippocampal Volume and Memory in Mood Disorders,” Biological Psychiatry 78 (2015): 270–277. 25641635 10.1016/j.biopsych.2014.12.013

[13] T. Wustenberg , M. Begemann , C. Bartels , et al., “Recombinant Human Erythropoietin Delays Loss of Gray Matter in Chronic Schizophrenia,” Molecular Psychiatry 16, no. 1 (2011): 26–36. 20479759 10.1038/mp.2010.51

[14] K. Yucel , M. C. Mckinnon , V. H. Taylor , et al., “Bilateral Hippocampal Volume Increases After Long‐Term Lithium Treatment in Patients With Bipolar Disorder: A Longitudinal MRI Study,” Psychopharmacology 195 (2007): 357–367. 17705060 10.1007/s00213-007-0906-9

[15] E. Cavedo , S. Galluzzi , M. Pievani , M. Boccardi , and G. B. Frisoni , “Norms for Imaging Markers of Brain Reserve,” Journal of Alzheimer's Disease 31 (2012): 623–633. 10.3233/JAD-2012-111817 22672878

[16] L. Yue , T. Wang , J. Wang , et al., “Asymmetry of Hippocampus and Amygdala Defect in Subjective Cognitive Decline Among the Community Dwelling Chinese,” Frontiers in Psychiatry 9 (2018): 226. 29942265 10.3389/fpsyt.2018.00226 PMC6004397

[17] N. T. Milne , R. S. Bucks , W. A. Davis , et al., “Hippocampal Atrophy, Asymmetry, and Cognition in Type 2 Diabetes Mellitus,” Brain and Behavior: A Cognitive Neuroscience Perspective 8 (2018): e00741. 10.1002/brb3.741 PMC5853633 29568674

[18] C. Geroldi , M. P. Laakso , C. Decarli , et al., “Apolipoprotein E Genotype and Hippocampal Asymmetry in Alzheimer's Disease: A Volumetric MRI Study,” Journal of Neurology, Neurosurgery, and Psychiatry 68 (2000): 93–96. 10601411 10.1136/jnnp.68.1.93 PMC1760588

[19] U. K. Haukvik , T. Mcneil , E. H. Lange , et al., “Pre‐ and Perinatal Hypoxia Associated With Hippocampus/Amygdala Volume in Bipolar Disorder,” Psychological Medicine 44 (2014): 975–985. 23803260 10.1017/S0033291713001529 PMC3936825

[20] S. R. Mathias , E. E. Knowles , J. W. Kent, Jr. , et al., “Recurrent Major Depression and Right Hippocampal Volume: A Bivariate Linkage and Association Study,” Human Brain Mapping 37 (2016): 191–202. 26485182 10.1002/hbm.23025 PMC4981180

[21] J. K. Wing , T. Babor , T. Brugha , et al., “SCAN: Schedules Fonr Clinical Assessment in Neuropsychiatry,” Archives of General Psychiatry 47 (1990): 589–593. 2190539 10.1001/archpsyc.1990.01810180089012

[22] B. Fischl , D. H. Salat , E. Busa , et al., “Whole Brain Segmentation: Automated Labeling of Neuroanatomical Structures in the Human Brain,” Neuron 33 (2002): 341–355. 11832223 10.1016/s0896-6273(02)00569-x

[23] J. L. Stein , S. E. Medland , A. A. Vasquez , et al., “Identification of Common Variants Associated With Human Hippocampal and Intracranial Volumes,” Nature Genetics 44 (2012): 552–561. 22504417 10.1038/ng.2250 PMC3635491

[24] M. Hamilton , “A Rating Scale for Depression,” Journal of Neurology, Neurosurgery, and Psychiatry 23 (1960): 56–62. 14399272 10.1136/jnnp.23.1.56 PMC495331

[25] R. C. Young , J. T. Biggs , V. E. Ziegler , and D. A. Meyer , “A Rating Scale for Mania: Reliability, Validity and Sensitivity,” British Journal of Psychiatry 133 (1978): 429–435. 10.1192/bjp.133.5.429 728692

[26] O. O. Aalen and S. Johansen , “An Empirical Transition Matrix for Non‐homogeneous Markov Chains Based on Censored Observations,” Scandinavian Journal of Statistics 5 (1978): 141–150.

[27] E. M. Arenaza‐urquijo and P. Vemuri , “Improving the Resistance and Resilience Framework for Aging and Dementia Studies,” Alzheimer's Research & Therapy 12 (2020): 1–4. 10.1186/s13195-020-00609-2 PMC7158381 32290864

[28] E. J. Canales‐rodriguez , E. Pomarol‐clotet , J. Radua , et al., “Structural Abnormalities in Bipolar Euthymia: A Multicontrast Molecular Diffusion Imaging Study,” Biological Psychiatry 76 (2013): 239–248. 24199669 10.1016/j.biopsych.2013.09.027

[29] Y. Sun , N. Hu , M. Wang , et al., “Hippocampal Subfield Alterations in Schizophrenia and Major Depressive Disorder: A Systematic Review and Network Meta‐Analysis of Anatomic MRI Studies,” Journal of Psychiatry & Neuroscience 48 (2023): E34–E49. 36750240 10.1503/jpn.220086 PMC9911126

[30] L. Frolich , O. Peters , P. Lewczuk , et al., “Incremental Value of Biomarker Combinations to Predict Progression of Mild Cognitive Impairment to Alzheimer's Dementia,” Alzheimer's Research & Therapy 9 (2017): 84. 10.1186/s13195-017-0301-7 PMC5634868 29017593

[31] J. Macoveanu , I. Meluken , L. V. Kessing , H. R. Siebner , M. Vinberg , and K. W. Miskowiak , “Hippocampal Subfield Morphology in Monozygotic Twins Discordant for Affective Disorders,” Neuropsychopharmacology 46 (2021): 561–568. 32620004 10.1038/s41386-020-0756-2 PMC8027865

[32] J. Macoveanu , K. Freeman , H. Kjaerstad , G. M. Knudsen , L. V. Kessing , and K. W. Miskowiak , “Structural Brain Abnormalities Associated With Cognitive Impairments in Bipolar Disorder,” Acta Psychiatrica Scandinavica 144 (2021): 379–391. 34245569 10.1111/acps.13349

[33] M. Keshavan , P. Lizano , and K. Prasad , “The Synaptic Pruning Hypothesis of Schizophrenia: Promises and Challenges,” World Psychiatry 19 (2020): 110–111. 31922664 10.1002/wps.20725 PMC6953570

[34] S. Kloiber , J. D. Rosenblat , M. I. Husain , et al., “Neurodevelopmental Pathways in Bipolar Disorder,” Neuroscience & Biobehavioral Reviews 112 (2020): 213–226. 32035092 10.1016/j.neubiorev.2020.02.005

[35] S. Vinogradov , M. V. Chafee , E. Lee , and H. Morishita , “Psychosis Spectrum Illnesses as Disorders of Prefrontal Critical Period Plasticity,” Neuropsychopharmacology 48 (2023): 168–185. 36180784 10.1038/s41386-022-01451-w PMC9700720

[36] J. A. Cobb , J. Simpson , G. J. Mahajan , et al., “Hippocampal Volume and Total Cell Numbers in Major Depressive Disorder,” Journal of Psychiatric Research 47 (2013): 299–306. 23201228 10.1016/j.jpsychires.2012.10.020 PMC3757567

[37] H. Bruehl , M. Rueger , I. Dziobek , et al., “Hypothalamic‐Pituitary‐Adrenal Axis Dysregulation and Memory Impairments in Type 2 Diabetes,” Journal of Clinical Endocrinology and Metabolism 92 (2007): 2439–2445. 17426095 10.1210/jc.2006-2540

[38] P. J. Lucassen , M. B. Muller , F. Holsboer , et al., “Hippocampal Apoptosis in Major Depression Is a Minor Event and Absent From Subareas at Risk for Glucocorticoid Overexposure,” American Journal of Pathology 158 (2001): 453–468. 11159183 10.1016/S0002-9440(10)63988-0 PMC1850286

[39] D. N. Alfarez , H. Karst , E. H. Velzing , M. Joels , and H. J. Krugers , “Opposite Effects of Glucocorticoid Receptor Activation on Hippocampal CA1 Dendritic Complexity in Chronically Stressed and Handled Animals,” Hippocampus 2008, no. 18 (2008): 20–28. 10.1002/hipo.20360 17708551

[40] B. Czeh and P. J. Lucassen , “What Causes the Hippocampal Volume Decrease in Depression? Are Neurogenesis, Glial Changes and Apoptosis Implicated?,” European Archives of Psychiatry and Clinical Neuroscience 257 (2007): 250–260. 17401728 10.1007/s00406-007-0728-0

[41] K. W. Miskowiak , N. Yalin , I. Seeberg , et al., “Can Magnetic Resonance Imaging Enhance the Assessment of Potential New Treatments for Cognitive Impairment in Mood Disorders? A Systematic Review and Position Paper by the International Society for Bipolar Disorders Targeting Cognition Task Force,” Bipolar Disorders 24 (2022): 615–636. 35950925 10.1111/bdi.13247 PMC9826389

