# Supplementary material for: Relation Between Brain Morphological Features and Psychiatric Hospitalization Risk in Major Depressive and Bipolar Disorders
Source: Acta Psychiatr Scand. 2025 Feb 20;151(6):689–97. doi: 10.1111/acps.13790 (PMC12045658; doi:10.1111/acps.13790)
Supplement: Supplementary file 1 — Data S1. [file ACPS-151-689-s001.docx]

**Supplementary File**

**Section S1: Information on ethical approval**

The procedures of the different original studies were in accordance with the ethical standards of the Danish Research Ethics Committee for the Capital Region (approval no. H-1-2010-039; H-16038506; H-16043370; H-16043480; H-7-2014-007; H-C-2008-092: H-3-2009-074), and Danish Data Protection Agency (2010-41-4710; RHP-2017-023; RHP-2017-020; 2012-58-0004; RHP-2015-023: 2008-41-2711; 2009-41-3676). The current National Health Register based study (i.e., BrainDrugs work-package 3) was approved by the Danish Data Protection Agency (Capital Region protocol number 2012-58-0004 [local journal number P-2021-110]). The work-package 3 of the BrainDrugs study involves conducting Danish register-based follow-up studies on already acquired deep phenotyping data (https://braindrugs.nru.dk/index.php/research).

**Section S2: The Danish national registers**

The Danish National Registers cover all residents living in Denmark and includes information on the date of birth, sex, death, migration, somatic and psychiatric diagnoses, hospitalizations, education, employment, and civil status, etc, and are updated at regular intervals. Data for each participant are anonymized by the data controllers at Statistics Denmark and are linked with the relevant registers. For instance, the Danish National Patient Register and the Danish Psychiatric Central Research Register contain data on diagnoses according to the ICD classification, psychiatric admissions, discharge dates, and treatments, and were used to determine if the patients were admitted to a psychiatric hospital anytime. Similarly, information on death and date of death was available from the Danish Register of Causes of Death.

**Section S3: Evaluation of verbal learning and memory scores in the sample**

Verbal learning and memory was assessed on the day of inclusion, using a broad battery of neuropsychological tests, from which composite z scores were calculated for the individual test batteries based on the mean and standard deviations of test scores from age and sex-matched healthy controls. The neuropsychological tests that were used to assess verbal learning and memory were the Screening in Cognitive Impairment in Psychiatry (SCIP) immediate and delayed verbal learning test, and the Rey Auditory Verbal Learning Test (RAVLT). For each participant, the z-scores from the available individual test batteries were then averaged to get a mean composite z score separately for the executive function and verbal memory cognitive domains (i.e., domain-specific z-scores). The domain-specific z scores are considered more reliable than the individual test scores due to reductions in measurement error. For the analysis performed herein, verbal learning and memory scores were dichotomized at z = −1, based on the International Society for Bipolar Disorders (ISBD) Targeting Cognition Task Force's definition of “clinically significant” cognitive impairment (1), and as done in our previous work examining cognitive risk factors of psychiatric hospitalization (2).

**Supplementary Table S1: Sensitivity analysis examining the association between hippocampal asymmetry and hospitalization risk using inverse of the probability of censoring weights (IPCW).**

| Time (years) | n.uncensored | max.IPCW | median.IPCW | OR | p,value |
| --- | --- | --- | --- | --- | --- |
| 1 | 273 | 1.19 | 1.19 | 1.01 | 0.86 |
| 2 | 192 | 1.87 | 1.87 | 0.94 | 0.43 |
| 3 | 151 | 2.67 | 2.67 | 0.84 | 0.07 |
| 4 | 151 | 2.67 | 2.67 | 0.81 | 0.02 |
| 5 | 150 | 2.70 | 2.70 | 0.81 | 0.02 |
| 6 | 147 | 2.79 | 2.79 | 0.86 | 0.08 |
| 7 | 137 | 3.19 | 3.19 | 0.84 | 0.04 |
| 8 | 111 | 5.09 | 2.32 | 0.86 | 0.13 |
| 9 | 81 | 16.57 | 1.13 | 0.58 | 0.05 |
|  |  |  |  |  |  |

“Time(years)” refers to the timepoints at which the survival is considered when evaluating the hazard ratio. The “n.uncensored” column refers to the number of individuals for which the hospitalization outcome is known at this timepoint. The “max.IPCW” and “median.IPCW” columns refer to the maximum and median weight used to re-weight observations with known hospitalization outcome when fitting the logistic model. OR represents the Odds Ratio. p-value at 10 years is not reported because the large IPCW makes the results unreliable.

The results tabulated in Table S1 show that for most of the time points, the significance of association between hippocampal asymmetry and the risk of future hospitalization was around p=0.05, and the odds ratio (OR) was estimated to be ~0.8.

**Section S4: Model covariates significantly associated with hospitalization risk**

The primary analysis indicated that in addition to higher rightward hippocampal asymmetry, higher intracranial volume was also associated with reduced risk of hospitalizations (HR=0.10, 95% CI: 0.01–0.80, p=0.03). In addition, hospitalization in the year preceding inclusion (HR=4.32, 95% CI: 2.24–8.34, p<0.001) and early years of inclusion (HR=1.06, 95% CI:1.01–1.11, p=0.03) were associated with increased risk of psychiatric hospitalizations. Exploratory analyses were consistent with the primary analysis, and indicated that intracranial volume, prior hospitalizations, and years of inclusion were also associated significantly with the risk of future psychiatric hospitalizations (p<0.047).

**References**

1. Miskowiak KW, Burdick K, Martinez‐Aran A, Bonnin C, Bowie C, Carvalho A, Gallagher P, Lafer B, López‐Jaramillo C, Sumiyoshi T. Assessing and addressing cognitive impairment in bipolar disorder: the International Society for Bipolar Disorders Targeting Cognition Task Force recommendations for clinicians. Bipolar disorders. 2018;20:184-194.

2. Sankar A, Ziersen SC, Ozenne B, Beaman EE, Dam VH, Fisher PM, Knudsen GM, Kessing LV, Frokjaer V, Miskowiak KW. Association of neurocognitive function with psychiatric hospitalization and socio-demographic conditions in individuals with bipolar and major depressive disorders. EClinicalMedicine. 2023;58.
